# Arguments for an additional long-lived intermediate in the photocycle of the full-length aureochrome 1c receptor: A time-resolved small-angle X-ray scattering study

**DOI:** 10.1063/1.5095063

**Published:** 2019-06-21

**Authors:** Saskia Bannister, Elena Böhm, Thomas Zinn, Thomas Hellweg, Tilman Kottke

**Affiliations:** 1 Physical and Biophysical Chemistry, Department of Chemistry, Bielefeld University, Universitaetsstr. 25, 33615 Bielefeld, Germany; 2 ESRF–The European Synchrotron, 71, Avenue des Martyrs, 38043 Grenoble Cedex 9, France

## Abstract

Aureochromes (AUREO) act as blue-light photoreceptors in algae. They consist of a light-, oxygen-, voltage-sensitive (LOV) domain and a DNA-binding basic region/leucine zipper. Illumination of the flavin cofactor in LOV leads to the formation of an adduct, followed by global structural changes. Here, we first applied UV/vis spectroscopy to characterize the photocycle of full-length aureochrome 1c (*Pt*AUREO1c) from the diatom *Phaeodactylum tricornutum*. With a time constant of 850 s and a quantum yield of 23%, *Pt*AUREO1c reveals a faster recovery time and a much lower sensitivity toward light than *Pt*AUREO1a, pointing to its role as a high light sensor *in vivo*. UV/vis spectroscopy offers details on the local recovery of the flavin chromophore. However, kinetic information on the global structural recovery of full-length AUREO or any other multidomain LOV protein is missing. This information is essential not least for the photoreceptors' applications as optogenetic devices. Therefore, we established a procedure to apply small-angle X-ray scattering on *Pt*AUREO1c in a time-resolved manner employing an in-house setup. In combination with UV/vis spectroscopy under similar conditions, we revealed a discrepancy between the recovery of the global protein structure and the adduct lifetime. Accordingly, we propose to supplement the photocycle by an intermediate state (I447), which decays with a time constant of about 800 s and prolongs the lifetime of the signaling state.

## INTRODUCTION

**FIG. 1.**
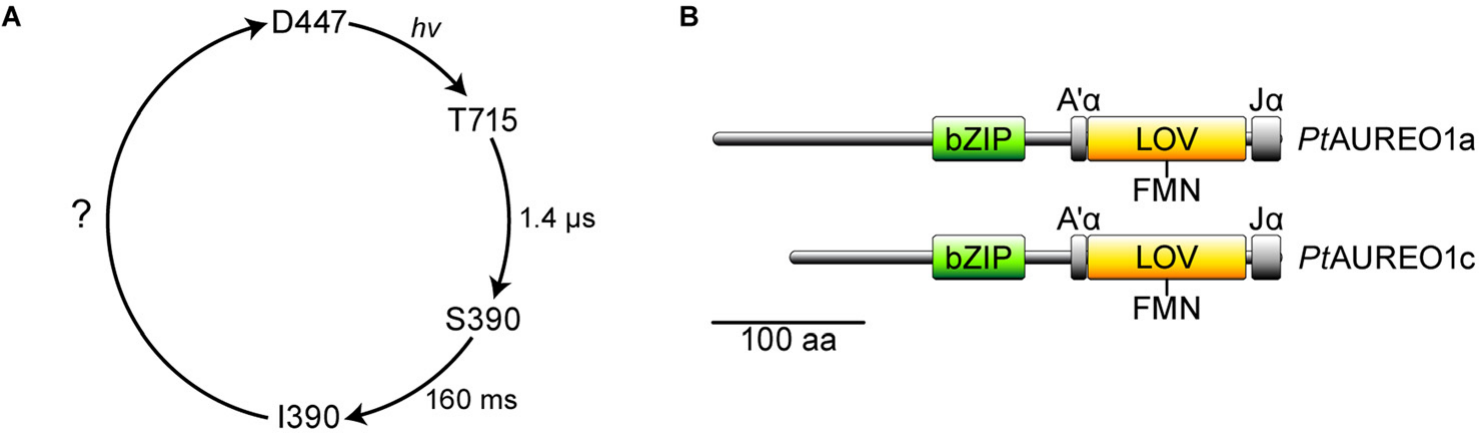
Photocycle kinetics and domain topology of aureochromes. (a) In the dark state (D447), flavin mononucleotide (FMN) is noncovalently bound to the LOV domain and has an absorption peak at 447 nm. Illumination with blue light leads to the formation of a triplet excited state (T715), which decays with a time constant of 1.4 *μ*s into a covalent adduct between the FMN and the LOV core.^49^ Conformational changes with a time constant of 160 ms occur in the bZIP domain, leading to the state I390.^9^ The cycle is completed when the adduct is cleaved and the initial conformation of the photoreceptor is restored. (b) Aureochromes are composed of a C-terminal LOV sensor domain with two flanking helices A′α and Jα, followed by an N-terminal bZIP effector domain and a long N-terminal extension. The N-terminal extensions in *Pt*AUREO1a and *Pt*AUREO1c differ in length.

Countless processes in all kingdoms of life are regulated by light, and these include the growth of plants (phototropism), the cell cycle of microorganisms, and the daily rhythm of animals, for instance. Therefore, it is not surprising that for the response to ambient light conditions, a variety of photoreceptors have evolved over time. Particularly noteworthy are photoreceptors that possess LOV (light-, oxygen-, voltage-sensitive) domains,^1,2^ which form a subgroup of the Per-ARNT-Sim^3^ (PAS) superfamily. LOV uses flavin mononucleotide (FMN) as a cofactor^4^ and undergoes a photocycle [Fig. 1(a)].^5^ In the dark, the oxidized flavin is bound noncovalently to the LOV core and an absorption maximum at ∼447 nm can be observed (D447). Illumination leads to the formation of a short-lived triplet excited state followed by the formation of a covalent bond between the flavin isoalloxazine ring and an adjacent, conserved cysteine residue of the LOV core. Adduct formation is accompanied by a blueshift of the absorption maximum to 390 nm (S390). The adduct thermally decays to the initial dark state within seconds to hours.^5^

LOV proteins fulfill a large variety of functions in cells, depending on the nature of the effector domain the LOV sensory domain may be linked to. One class of LOV proteins are aureochromes (AUREO), which were first encountered in the alga *Vaucheria frigida*.^6^ As their effector domain consists of a DNA-binding basic region/leucine zipper (bZIP),^7^ their role as a light-regulated transcription factor was suggested.^6^ The most striking feature of aureochromes is their unusual domain topology. In contrast to most other LOV proteins,^8^ the sensory domain is located at the C-terminus, whereas the bZIP effector domain is located at the N-terminus followed by a long N-terminal extension [Fig. 1(b)].^6^ The question, which underlying allosteric regulation mechanism results in a signal transduction from the C-terminus to the N-terminus, has been studied extensively in recent years.^9–12^ These investigations have targeted two homologous members of the aureochrome class 1, *Vf*AUREO1 from *V. frigida* and *Pt*AUREO1a from the diatom *Phaeodactylum tricornutum*, whereas very little is known about members of two other classes comprising *Pt*AUREO1b and *Pt*AUREO1c, respectively.^10,13^

Small angle X-ray scattering (SAXS) is a powerful technique to study light-dependent structural changes in photoreceptors under near-native conditions in solution. Investigations on LOV domains with different flanking elements have led to low-resolution models, which allow conclusions on oligomeric states and flexible regions.^14–16^ But SAXS is not limited to small, one-domain structures, it is also applicable to multidomain proteins that are highly challenging to crystallize. Previously, first insight into the light-dependent arrangement of domain positions in phototropin was gained, which consists of two LOV and a kinase domain.^17^ Moreover, the LOV-STAS (sulfate transporter and antisigma factor) domain assembly of the YtvA photoreceptor has been clarified.^18^ Solution SAXS experiments on the N-terminally truncated *Pt*AUREO1a, which comprises the LOV and the bZIP domain, have contributed to the development of two models, which describe the allosteric regulation mechanisms of aureochromes.^10,11^ Full-length *Pt*AUREO1a, however, has been found unsuitable for SAXS studies.^11^

Even though SAXS experiments under steady-state conditions give essential insight into the activation of photoreceptors, there is still considerable room for speculation on the chronological sequence of processes that lead to the transition from the dark to the lit state and back. Time-resolved X-ray scattering can overcome this issue and has been achieved on LOV proteins. For instance, X-ray scattering experiments in the nanosecond-to-second time range revealed intermediate conformational states of an artificial LOV-histidine kinase.^19^ Moreover, dimerization of Vivid, a LOV domain with N-terminal cap, was evaluated by time-resolved photocoupled SAXS in the millisecond-to-second time range.^20^ Even the slow transition back to the dark form in the minute range of a short LOV protein^21^ has been the objective of time-course SAXS.^22^

Here, we characterize *Pt*AUREO1c [Fig. 1(b)] for the first time with respect to its photocycle kinetics and quantum yield of conversion and highlight its fundamental differences in direct comparison to *Pt*AUREO1a. We found that full-length *Pt*AUREO1c is amenable to SAXS experiments, which allowed us to study the recovery of the global structure after illumination using time-resolved SAXS (TR-SAXS). Our data indicate that the overall protein structure does not revert concomitantly with breakage of the flavin adduct in LOV but requires additional time to complete. Accordingly, results from UV/vis spectroscopy do not reflect the overall restoration of the aureochrome's dark state. This finding on the global recovery time fills a gap in the photocycle of aureochrome [Fig. 1(a)], which is also of relevance for other LOV-based photoreceptors.

## MATERIALS AND METHODS

### Expression plasmids

Detailed information on the expression plasmids encoding *Pt*AUREO1a and *Pt*AUREO1c [Protein-IDs 49116 and 56742, respectively, Joint Genome Institute (JGI) database] from the diatom *Phaeodactylum tricornutum* were provided previously.^10^

### Protein expression and purification

For characterization by UV/vis spectroscopy, the expression of *Pt*AUREO1a and its purification were carried out as described previously.^10^ The same procedure was followed for *Pt*AUREO1c applied in UV/vis kinetic experiments. The samples were obtained in 50 mM phosphate buffer with pH 8.0 containing 300 mM NaCl and 20% glycerol.

For SAXS experiments, UV/vis absorption spectroscopy, and UV/vis kinetic experiments under similar conditions to those in SAXS, *Pt*AUREO1c was heterologously expressed in *Escherichia coli* BL21 (DE3) pLysE (Invitrogen). The cells were grown in DYT medium containing 30 *μ*g/ml kanamycin at 37 °C until an OD_600_ of 0.55 was reached. Then, the temperature was set to 18 °C. At an OD_600_ of about 0.85, protein expression was induced with 10 *μ*M isopropyl-*β*-d-thiogalactopyranoside (IPTG). The cells were incubated for 20 h and pelleted by centrifugation afterward. The pellets were resuspended in 350 mM Tris buffer, pH 8, containing 300 mM NaCl and 20 mM imidazole. A protease inhibitor tablet (cOmplete Ultra EDTA-free, Roche) and DNase were added to the solution. The cells were lysed with a French press (SLM Aminco) at 1000 psig and centrifuged at 108 000×g and 4 °C. A Co^2+^-enriched His-Bind resin column (Novagen) was loaded with the supernatant and washed with 50 mM Tris buffer containing 20 mM imidazole and 40 mM imidazole successively. The protein was eluted with the same buffer containing 250 mM imidazole. Samples were concentrated in 30 kDa cut-off centrifugal filters (Amicon Ultra-15, Merck Millipore). To enhance the chromophore occupancy, the proteins were reconstituted with up to the five-fold amount of FMN overnight at 4 °C in the dark. Excess FMN was partially removed as the samples were concentrated in 30 kDa cut-off centrifugal filters. Finally, *Pt*AUREO1c was subjected to purification via size exclusion chromatography (SEC) on a HiLoad Superdex200pg 16/60 column (GE Healthcare) at 4 °C in the dark. The protein was obtained in 10 mM Tris buffer with pH 8.0 at 10 °C containing 300 mM NaCl. Fractions revealing a chromophore occupancy ≥60% were merged and concentrated in 30 kDa cut-off centrifugal filters. The samples were shock-frozen in liquid nitrogen and stored at −80 °C in the dark until use. After thawing, *Pt*AUREO1c was obtained as a homogenous sample without aggregates as confirmed by performing an analytical SEC using a Superdex200 10/300 GL column (GE Healthcare) at 4 °C in the dark (Fig. S1 in the supplementary material).

### UV/vis experiments

UV/vis spectra of *Pt*AUREO1a and *Pt*AUREO1c were recorded using a Shimadzu UV-2450 spectrometer. The concentrations of the samples were obtained by taking the extinction coefficients of free FMN at 280 nm and 450 nm,^23^ *Pt*AUREO1a at 280 nm (17 210 M^−1 ^cm^−1^), and *Pt*AUREO1c at 280 nm (12 090 M^−1 ^cm^−1^) into account. The extinction coefficients of *Pt*AUREO1a and *Pt*AUREO1c were calculated.^24^

Kinetic experiments were performed in a quartz cell (Hellma Analytics) with a path length of 1 cm by monitoring the recovery of absorbance at 447 nm under rigorous darkness after illuminating the samples with a blue light-emitting diode (LED). The proteins obtained in phosphate buffer were exposed to a light pulse [455 nm, 20 mW cm^−2^, 20 nm FWHM (full width at half maximum), Philips Lumileds] for 500 ms. The experiments on *Pt*AUREO1a and *Pt*AUREO1c were conducted at 20 °C. Both samples had an absorption of 0.4 at 448 nm.

Kinetic experiments under SAXS conditions were performed in a quartz cell (Hellma Analytics) with a path length of 1 mm. *Pt*AUREO1c (9.9 mg/ml) in Tris buffer at 11.4 °C was illuminated for 60 s with a blue LED (474 nm, 56 mW cm^−2^, 21 nm FWHM, Philips Lumileds). The quality of the samples was controlled by recording absorption spectra.

**FIG. 2.**
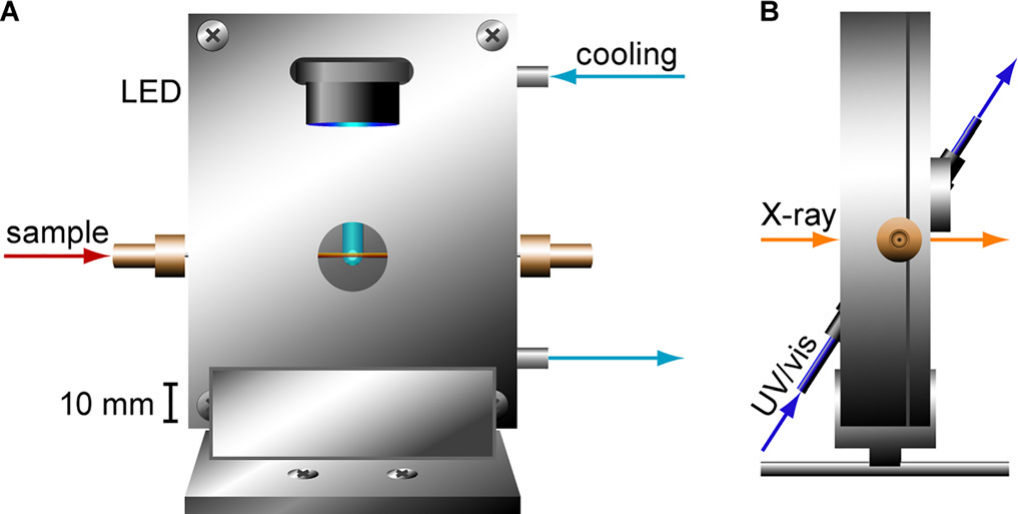
Sample environment design for SAXS experiments on light-sensitive proteins. (a) Front view of the flow-through sample chamber with an inserted Kapton capillary and an LED for illumination of the sample. The temperature is regulated by a circulating water bath. (b) Side view of the SAXS sample environment. The front plate is exchanged to include a lens and light fibers to a light source and a detector in order to record UV/vis spectra of the sample within the capillary.

A HR2000+ high resolution spectrometer with a DH-2000-BAL UV-Vis-near infrared light source (Ocean Optics) was utilized to record UV/vis absorption spectra of *Pt*AUREO1c (11.2 mg/ml) in the Kapton capillary of a homemade flow-through sample chamber, which was developed for SAXS experiments [Fig. 2(b)]. After an absorption spectrum was recorded under rigorous darkness, the sample was illuminated for 2 min with a blue LED (474 nm, 56 mW cm^−2^, 21 nm FWHM, Philips Lumileds) and the absorption spectrum of its lit state was recorded. The temperature was 11.4 °C.

### SAXS experiments

For all SAXS experiments, a homemade flow-through sample chamber with an inserted Kapton capillary (inner diameter: 1 mm, wall thickness: 25 *μ*m; GoodFellow) and an LED (474 nm, 56 mW cm^−2^, 21 nm FWHM, Philips Lumileds) was applied [Fig. 2(a)]. The temperature in the sample chamber was kept at 11.4 °C by a circulating water bath.

The synchrotron SAXS experiments on *Pt*AUREO1c (12.9 mg/ml) were performed at the ESRF SAXS beamline ID02, Grenoble, France^25^ with an X-ray energy of 12.46 keV and a sample-to-detector distance of 1 m. This setup allowed to access a scattering vector (of magnitude *q*) range of 0.08–7.74 nm^−1^ where all important features of *Pt*AUREO1c could be identified. The magnitude of the scattering vector ***q*** is given by 4π sin(θ)/*λ* with 2θ being the scattering angle and *λ* being the incident X-ray wavelength (0.995 Å).

Fifteen frames were recorded with an exposure time of 50 ms each. Before insertion into the capillary, the samples were centrifuged for 15 min at 4 °C and 18 500×g. For the investigation of the photoreceptor's dark state, experiments were conducted under rigorous darkness. For its lit state, *Pt*AUREO1c was exposed to blue light for 120 s in the sample chamber prior to the data acquisition and further illuminated throughout the data collection.

SAXS kinetic experiments were conducted under rigorous darkness on an in-house Xeuss (Xenocs) system, which comprises a GeniX 3D Cu Ultra Low Divergence CuK_α_ X-ray source (*λ* = 1.541 Å; Xenocs) and a hybrid pixel detector (Pilatus 300 K 20 Hz, Dectris). A sample-to-detector distance of 0.534 m resulted in a *q*-range of 0.26–5.66 nm^−1^.

Before use, the samples were thawed on ice and stored overnight at 4 °C in the dark. The samples were centrifuged for 30 min at 4 °C and 21 400×g directly before insertion into the capillary under red safety light. Experiments on *Pt*AUREO1c with concentrations of 9.9 mg/ml and 10.8 mg/ml were conducted in the dark for 4 h (48 frames) followed by three subsequent kinetic measurements on the same samples. Each kinetic experiment was initiated by illumination for 60 s. To guarantee full recovery back to the protein's dark state, 400 min (80 frames) lay between two illumination sequences. A total of 288 SAXS frames, with a measuring time of 300 s each, were collected in one kinetic experiment on the in-house SAXS system.

### SAXS data treatment

To obtain one-dimensional scattering profiles *I*(*q*) in absolute units, i.e., cm^−1^, the measured two-dimensional scattering patterns were azimuthally averaged and normalized according to standard procedures using ESRF SAXS programs^26^ and ESRF *SAXSutilities* software packages.^25^ Tools from these packages were also used for further data processing including background subtraction, absolute scaling, and averaging of the normalized data presented here. Water was applied as a standard for the synchrotron data,^27^ whereas glassy carbon type 2 was used for the laboratory SAXS beamline.^28^ The frames recorded at the synchrotron were checked for possible radiation damage (Fig. S2), and two frames were employed for averaging.

For better statistics of the SAXS kinetic data, two subsequent 300 s frames were added up to 600 s frames. Global time information was extracted by singular value decomposition (SVD) with a home-written SVD program for MATLAB (MathWorks) according to *A_exp_* = *U*·*S*·*V*^T^. SVD was performed considering five components and neglecting regions of *q *≥* *2.1 nm^−1^ dominated by noise. Based on the equation for consecutive first-order reactions,^29^ the weighted first component *V_i1_·S_11_* was fitted by

$${\left(V_{i1}\cdot S_{11}\right)}_{t}=A\cdot (1-\frac{1}{k_{I447}-k_{I390}}\cdot \left(k_{I447}\cdot e^{-k_{I390}\cdot t}-k_{I390}\cdot e^{-k_{I447}\cdot t}\right))+{\left(V_{i1}\cdot S_{11}\right)}_{0},$$ (1)

with the amplitude *A*, the rate constant for adduct decay *k_I_*_390_, which was determined by UV/vis spectroscopy, and an additional rate constant *k_I447_*, which is observed in the SAXS data.

### Numerical simulation of receptor activation

**FIG. 3.**
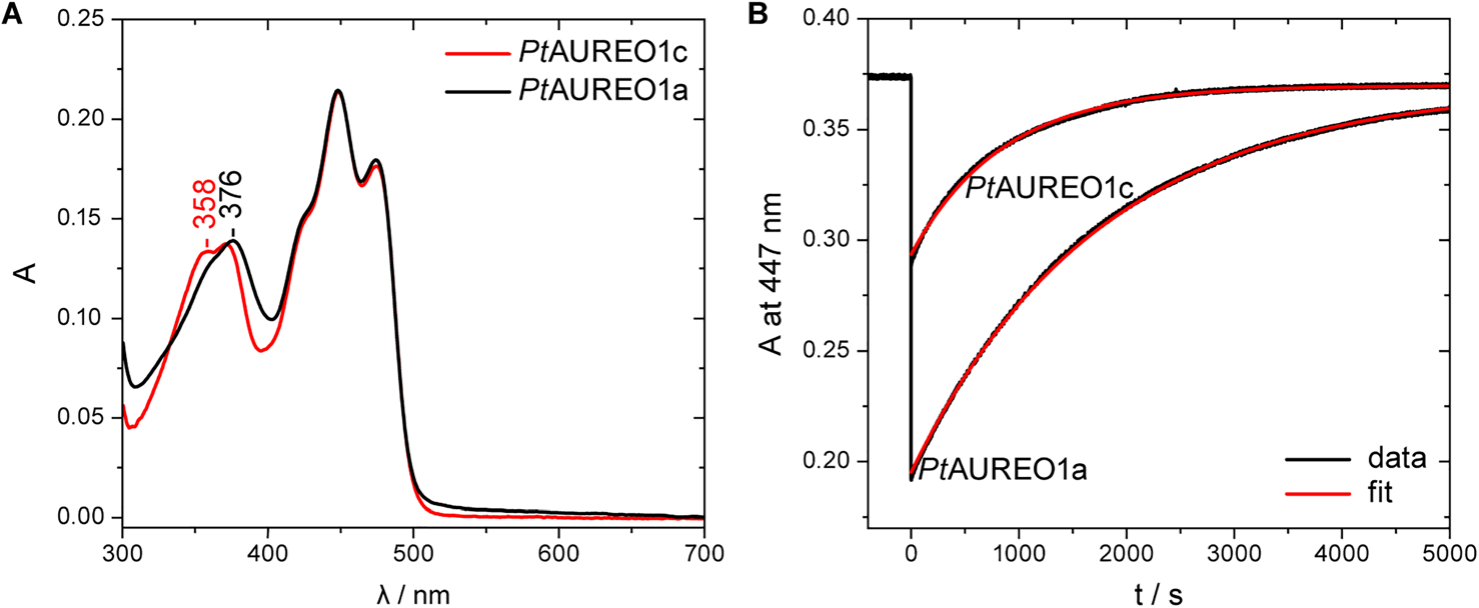
UV/vis absorption spectrum and recovery kinetics of full-length *Pt*AUREO1c in comparison to *Pt*AUREO1a. (a) The absorption spectrum of *Pt*AUREO1c shows the typical features of a flavoprotein with bands in the UVA and blue region. The difference in band shapes at around 358 nm compared to *Pt*AUREO1a is attributed to a different interaction in the flavin binding pocket. (b) The recovery of oxidized flavin in *Pt*AUREO1c after illumination with blue light was analyzed to result in *τ* = 850 s at 20 °C. Under identical conditions, *Pt*AUREO1a shows a significantly higher conversion by light and a slower recovery in the dark (*τ* = 1750 s).

At the photostationary state, the concentration of activated receptor *AUREO_lit_* is given by^30^

$$\left[{\mathrm{AUREO}}_{\mathrm{lit}}\right]=-k\;\left[{\mathrm{AUREO}}_{\mathrm{lit}}\right]+Ф\cdot F\cdot \int \varepsilon \left(\lambda \right)\rho \left(\lambda \right)d\lambda \cdot [{\mathrm{AUREO}}_{\mathrm{dark}}],$$ (2)

with the rate constant of the thermal recovery *k*, the quantum yield *Φ*, the photon flux *F*, the extinction coefficient *ε*, and the normalized quantum emission spectrum of the sun *ρ*. The numerical simulation was performed in MATLAB using the solar spectrum AM1.5g, an extinction coefficient at 450 nm of 12 500 M^−1 ^cm^−1^,^23^ and the spectra shown in Fig. 3 at 300–700 nm.

## RESULTS

### Recovery kinetics and quantum yield by UV/vis spectroscopy

The subfamily of *Pt*AUREO1c within the aureochromes is much less investigated than the homologs of *Pt*AUREO1a. Accordingly, the absorption spectrum of full-length *Pt*AUREO1c was recorded in direct comparison to that of *Pt*AUREO1a [Fig. 3(a)]. In the dark, both proteins reveal an identical behavior in the region of the first electronic transition of the protein-bound, oxidized flavin with an absorption maximum at 448 nm. However, the absorptions in the UV/A region of the spectrum deviate from each other. The spectrum of *Pt*AUREO1a has a single maximum at 376 nm, whereas *Pt*AUREO1c reveals a hypsochromic shift with two absorption maxima at 358 nm and 371 nm. This difference has been attributed previously for phototropin LOV1 and LOV2 domains to be caused by the presence of a threonine residue in the flavin-binding pocket close to the isoalloxazine methyl group.^31^ Accordingly, *Pt*AUREO1a (with T255 conserved) exhibits a LOV2-like spectrum and *Pt*AUREO1c (with S199 instead at this position) a LOV1-like spectrum, which allows us to differentiate the two subfamilies of aureochromes using UV/vis spectroscopy.

Next, the recovery of *Pt*AUREO1c after illumination was studied by UV/vis kinetic experiments at 447 nm. For comparison, studies were performed on *Pt*AUREO1a and *Pt*AUREO1c under identical conditions at 20 °C [Fig. 3(b)]. Both photoreceptors were obtained in 50 mM phosphate buffer with 300 mM NaCl and 20% glycerol at pH 8. A baseline was recorded for 500 s in the dark. Subsequent illumination with blue light for 500 ms led to the formation of the flavin-cysteine adduct with a strongly reduced extinction coefficient at 447 nm.^32^ The flavin recovery was recorded in the dark, and monoexponential functions were fitted to the datasets. Time constants of 850 s (R^2^ = 0.998) for *Pt*AUREO1c and 1750 s (R^2^ = 0.9995) for *Pt*AUREO1a were obtained. The data indicate that the recovery of *Pt*AUREO1c is two times faster than that of *Pt*AUREO1a. A further prominent feature is that under identical conditions, *Pt*AUREO1c shows a much smaller sensitivity toward light than *Pt*AUREO1a, which can be seen in the bleaching differences of their chromophore's absorptions at 447 nm directly after illumination. To quantify this difference, we determined the quantum yields for adduct formation of the two photoreceptors in reference to the known value of 60% of phototropin-LOV1 from *Chlamydomonas reinhardtii* (Fig. S3).^33,34^ This procedure led to values of 23% for *Pt*AUREO1c and 64% for *Pt*AUREO1a and therefore confirms that *Pt*AUREO1c's sensitivity toward light is about three times lower than the one of *Pt*AUREO1a.

### Recovery of the protein structure by SAXS experiments

UV/vis spectroscopy allows the determination of the time constant for the flavin chromophore recovery. However, information on the recovery of the protein structure is missing and hardly amenable. SAXS experiments on the biomolecule in solution may provide a valuable approach to determine the protein recovery time.

**FIG. 4.**
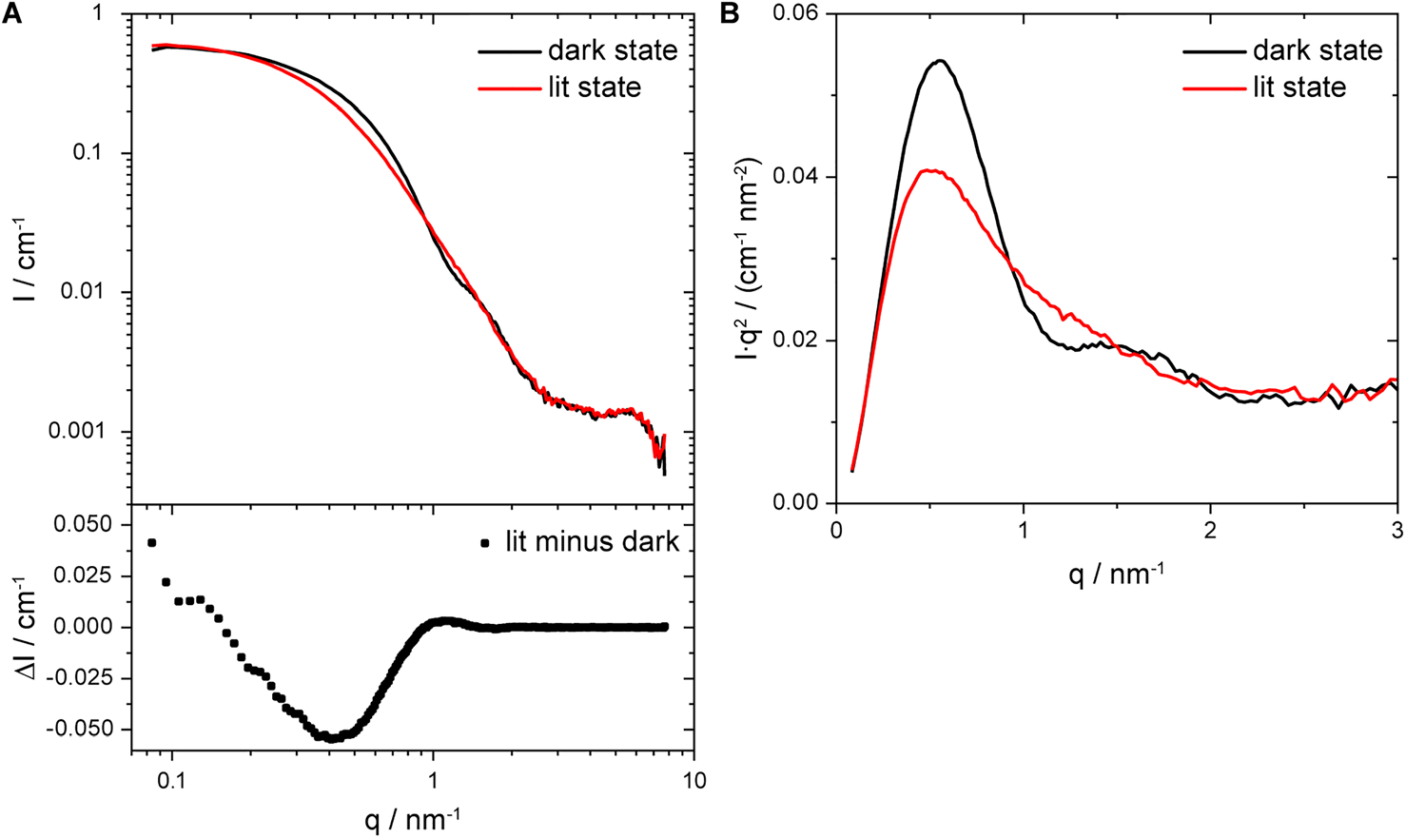
SAXS experiment on *Pt*AUREO1c in solution at the ESRF ID02 synchrotron beamline. (a) The scattering profiles (top) of the photoreceptor's dark state and the lit state after illumination for 120 s with blue light and their corresponding difference profile (bottom) are presented. (b) The Kratky plot demonstrates that illumination leads to characteristic and prominent changes in the scattering profiles, which indicate light-dependent changes in the structural envelope of *Pt*AUREO1c. The SAXS experiments were conducted at 11.4 °C. The protein concentration was 12.9 mg/ml.

For an initial characterization, we performed SAXS experiments on full-length *Pt*AUREO1c at the ESRF ID02 synchrotron beamline. The scattering profile of the photoreceptor changed upon illumination with blue light (λ = 474 nm) compared to the dark state scattering profile, which indicates that structural changes occurred [Fig. 4(a), top]. The largest, resolvable light-dependent changes range up to a *q*-range of 2.5 nm^−1^, as is demonstrated by the difference profile of the synchrotron data [Fig. 4(a), bottom]. The intensities *I*_0_ at small *q* were determined by using the Guinier approximation. *I*_0_ of the lit state is by a factor of 1.05 larger than the one from the dark state. This small change cannot be attributed to an oligomerization of the photoreceptor. In the Kratky presentation, the scattering data are weighted by *q*^2^ and thereby enable a closer view on intermediate *q*-regions, where the differences are most prominent [Fig. 4(b)]. In the initial dark state, the protein reveals a bell-shaped feature with a peak at 0.55 nm^−1^. After illumination, the peak position is slightly shifted to lower *q* (0.52 nm^−1^) and the scattering profile becomes flatter [Fig. 4(b)]. We conclude that the sample is amenable to investigation by SAXS and that the observed light-induced process is identified as an internal structural rearrangement as opposed to a simple change in oligomerization. A more detailed evaluation of the structural changes by deriving models from the scattering curves will be provided elsewhere. Here, we focus on the kinetics of the global structural changes. Synchrotron radiation is not the right tool for our continuous kinetic experiments on such photoreceptors because of the beam damage (Fig. S2) in conjunction with the long recovery times of *Pt*AUREO1c. By accepting a loss of brilliance of the X-ray radiation source, we used an in-house laboratory beamline instead.

To ensure the success of these investigations, it is essential that rigorous dark and light conditions are guaranteed. In order to prevent the conversion of the sample by ambient light during the loading of the capillary, a blackbox surrounding the sample environment was installed. Due to the long duration of the SAXS kinetic experiments of 24 h, a capillary material was required that impairs sample stability as little as possible and exhibits a low X-ray absorption. Therefore, a Kapton capillary was preferred over a conventional quartz capillary. As a positive side effect, the applied capillary blocks light below 460 nm and therefore offers extra protection of the sample against light. However, compared to the quartz glass, Kapton is flexible and it shows distinct features in its X-ray diffraction pattern. This may cause problems with background subtraction of weak scattering samples such as proteins in solution. Therefore, special care had to be taken when handling the Kapton capillary.

**FIG. 5.**
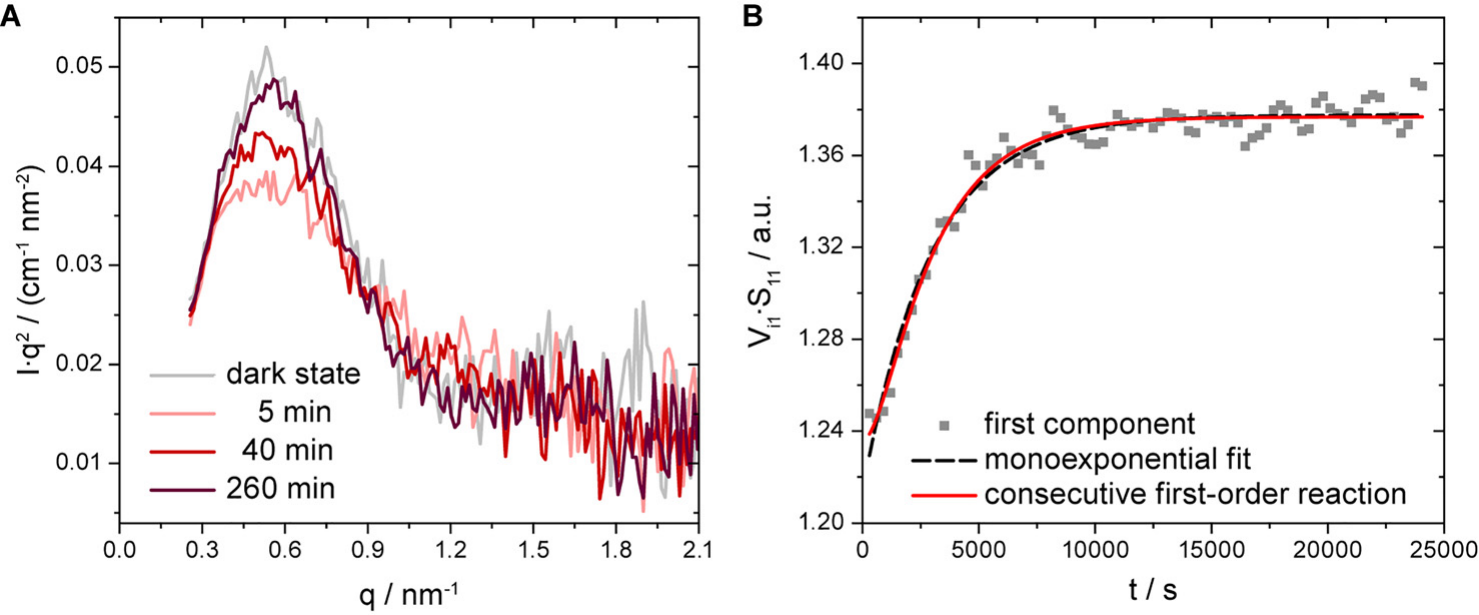
TR-SAXS experiment on *Pt*AUREO1c in solution. (a) The scattering profiles of the initial dark state and three exemplary time points after illumination of *Pt*AUREO1c for 60 s with blue light are presented as Kratky plots averaged over three consecutive experiments. The data demonstrate that the characteristic changes in the scattering profile by light are reversible over time and indicate the dark recovery of the protein envelope. (b) The scattering profiles were analyzed by singular value decomposition (SVD) restricted to a *q*-range of 0.26 to 2.1 nm^−1^. Global kinetic information was extracted from the most significant, first SVD component by fitting with a monoexponential function yielding a mean time constant of 2966 s of two independent datasets comprising three kinetic experiments each [see Fig. 6(b) for error bars]. As the global structural recovery times deviate significantly from the time constant determined for the adduct decay, the data were fit additionally by a model for a consecutive first-order reaction [Eq. (1)]. The experiments were conducted for protein concentrations of 10.8 mg/ml and 9.9 mg/ml in the dark at 11.4 °C and 300 s frames were recorded. Two subsequent frames were added up to 600 s frames.

Three recovery kinetics of *Pt*AUREO1c at 9.9 mg/ml and 10.8 mg/ml, respectively, were recorded over a time range of several hours in the dark at 11.4 °C. The low temperature was chosen to stabilize the protein for the long duration of the experiments. Each experiment was initiated by illuminating the protein solution for 60 s with a blue LED that was inserted above the capillary in the sample chamber. The long illumination was applied to maximize the change in the scattering signal for kinetic evaluation. Since no evident aggregation was observed, the three datasets of each concentration were averaged. The changes in the scattering profile of *Pt*AUREO1c are best displayed in a Kratky plot [Fig. 5(a)]. These changes strongly resemble those obtained in the synchrotron experiments [Fig. 4(b)]. After some time in the dark, the initial peak structure is observed again, which indicates that the protein has returned to its dark-adapted state and therefore the structural changes are fully reversible.

**FIG. 6.**
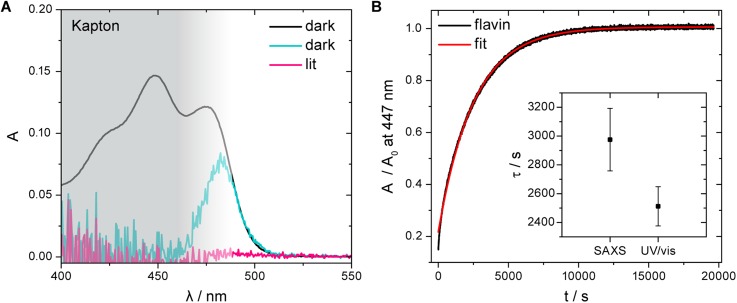
UV/vis spectroscopy on *Pt*AUREO1c under conditions of the SAXS experiments. (a) In the absorption spectra of *Pt*AUREO1c (11.2 mg/ml) in a Kapton capillary, only the low energy flank of the absorption band can be observed (blue line), as the Kapton capillary with an inner diameter of 1 mm and a wall thickness of 25 *μ*m used for the SAXS kinetic experiments effectively blocks light below 460 nm. The illumination for 120 s at 474 nm results in a complete loss of absorption indicating full conversion (pink line). The spectrum in a quartz cuvette is shown for comparison (black line). (b) The sample was illuminated for 60 s with blue light similar to the SAXS kinetic experiments and the recovery of the flavin absorbance was monitored in the dark. An averaged time constant of 2512 s was extracted by analysis with a monoexponential function. The experiments were performed at 11.4 °C. The concentration of the protein solution was 9.9 mg/ml. The inset shows the mean value ± the maximal deviation of three experiments for the UV/vis kinetics and for the SAXS data [see Fig. 5(b)]. This evaluation clearly demonstrates the significance of the results and the conclusions drawn.

To evaluate the recovery kinetics despite the considerable noise level in the experiments, singular value decomposition (SVD) with five components was performed on the unweighted datasets restricted to a *q*-range of 0.26 to 2.1 nm^−1^, considering the full time range of up to 400 min. Matrices *U*, *S*, and *V* containing information on the scattering profiles, the singular values, and the time courses, respectively, were obtained.^35^ The comparisons between the weighted scattering profiles (*U_ij_*·*S_jj_*) [Fig. S4(a)] and between the weighted time courses (*V_ij_*·*S_jj_*) [Fig. S4(b)] of each SVD component *j* indicate that the first component is the dominating one and carries essentially all information. We computed the matrices *A_SVD,m_* representing the reconstructed time-resolved scattering data by using the relation *A_SVD_*=*U*·*S*·*V*^T^ and calculated the average relative deviation to the experimental data matrix *A_exp_* in dependence of the number of components *m*. The comparison emphasizes that no significant improvement is achieved with more than one component (Fig. S5), and therefore, global kinetic information was extracted from the most significant, first component by fitting a monoexponential function to the weighted time course (*V_i1_*·*S_11_*) [Fig. 5(b)]. Three sequential experiments on the same sample showed a mean time constant of 2975 s with a maximal deviation of 7.3% [Fig. 6(b), inset]. To improve the signal-to-noise ratio, these three experiments were averaged and evaluated again by SVD [Fig. 5(b)]. From two independent datasets of such three experiments, we obtained a mean time constant of 2966 s with a deviation of 0.2% from the two individual values for the recovery of the protein envelope after illumination.

To evaluate the results obtained using X-ray scattering, UV/vis spectroscopy was performed under SAXS conditions. The absorption spectra before and after illumination for 120 s in the Kapton capillary provide evidence for a complete conversion of the sample [Fig. 6(a)]. For direct comparison of the flavin adduct decay and the protein structural recovery, we performed time-resolved UV/vis spectroscopy on *Pt*AUREO1c in a cuvette with a 1 mm path length at 11.4 °C after the sample was illuminated by blue light for 60 s. Absorption spectra between subsequent kinetic experiments demonstrate that the sample quality was little impaired throughout the experiment despite the long illumination time (Fig. S6). An average time constant of 2512 s with a maximal deviation of 5% was extracted by fitting monoexponential functions to the datasets [Fig. 6(b)]. This time constant is significantly higher than 850 s determined at 20 °C [Fig. 3(b)], which is attributed to the difference in temperature. The activation barrier in the Arrhenius equation corresponding to such a difference in time constants would be 87 kJ/mol, which is in agreement with the 52–100 kJ/mol found for other LOV domains.^36,37^ Additional influences on the flavin recovery such as protein concentration, duration of illumination, spectrum, and intensity of the LED cannot be excluded, which is why all these parameters were chosen here [Fig. 6(b)] to be the same as in the SAXS experiments.

**FIG. 7.**
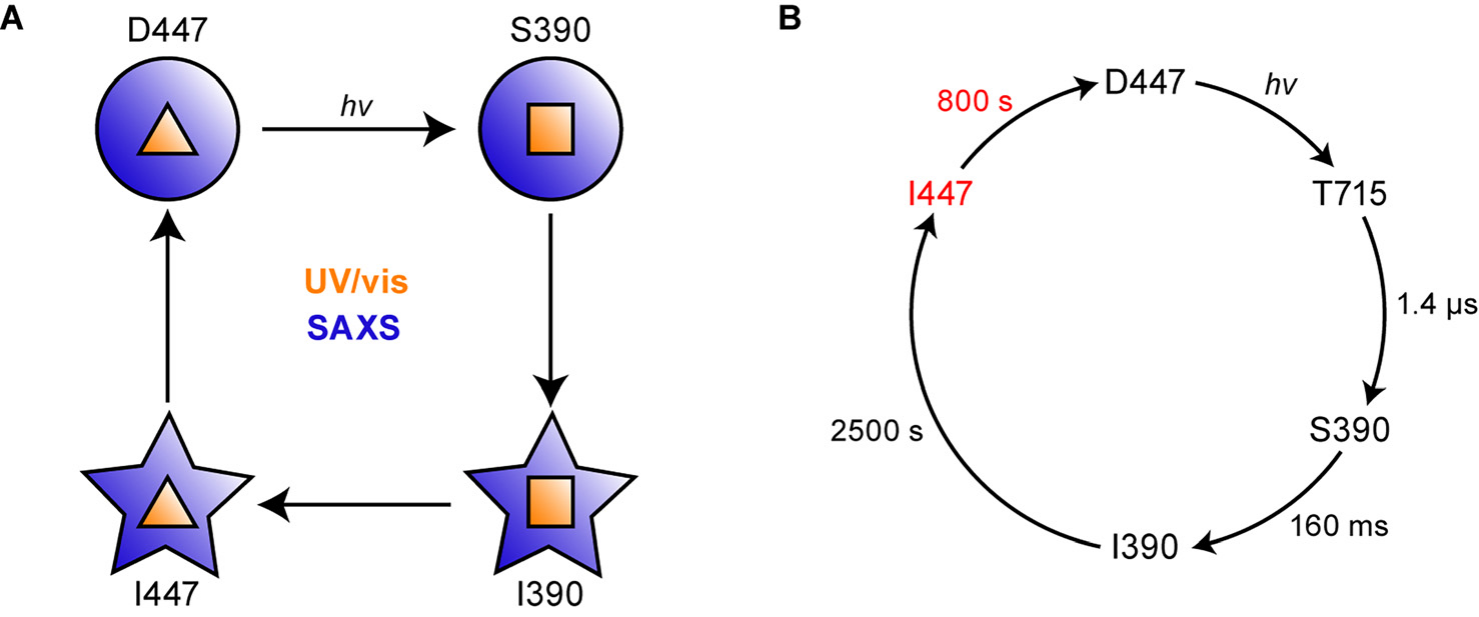
A new intermediate I447 augments the photocycle of aureochrome. (a) Time-resolved SAXS and UV/vis spectroscopy on *Pt*AUREO1c reveal a discrepancy between the times of global structural recovery (blue) and the recovery of the flavin's oxidized state (yellow). (b) The comprehensive photocycle of aureochrome includes states I390 and I447 which are silent to UV/vis spectroscopy. Illumination of the photoreceptor leads to the formation of the flavin adduct of LOV (S390), followed by conformational changes in bZIP (I390). Cleavage of the adduct promotes a local structural recovery in the vicinity of flavin but does not allow yet for a full reversal of the global conformation (I447). The cycle is only closed when the initial conformation of the photoreceptor is restored (D447).

A direct comparison between the time constants obtained by UV/vis spectroscopy and by SAXS demonstrates that the recovery of the global protein structure is slower than the initiating breakage of the flavin-cysteine adduct. Although the time constants are in the same order of magnitude, the fit to the SAXS-derived kinetics improved significantly by converging to the slower time constant (Fig. S7). Moreover, this discrepancy is beyond the limits of the maximal errors of the experiments [Fig. 6(b), inset], which points to the presence of an additional process. The simplest kinetic model to account for the discrepancy includes a spectrally silent intermediate I447 (Fig. 7). As a consequence, a monoexponential fit to the SAXS data is not applicable anymore, because such a function can only be applied to a single (pseudo) first-order reaction. Instead, Eq. (1) was applied to determine the decay rate constant *k_I_*_447_ of the intermediate under the assumption of consecutive first-order reactions for the back reaction of *Pt*AUREO1c.^29^ It should be noted that the accuracy of the fit is limited by the similar order of the time constants of the adduct decay and the overall structural recovery and by the data quality which is directly related to the sample concentration. By fixing the adduct decay rate constant *k_I_*_390_ = (2512 s)^−1^, which was determined by UV/vis spectroscopy, *k*_I447_ ∼ (800 s)^−1^ (at 10.8 mg/ml) was determined for the additional intermediate state I447.

## DISCUSSION

### The rationale behind the presence of two similar photoreceptors in the same organism

**FIG. 8.**
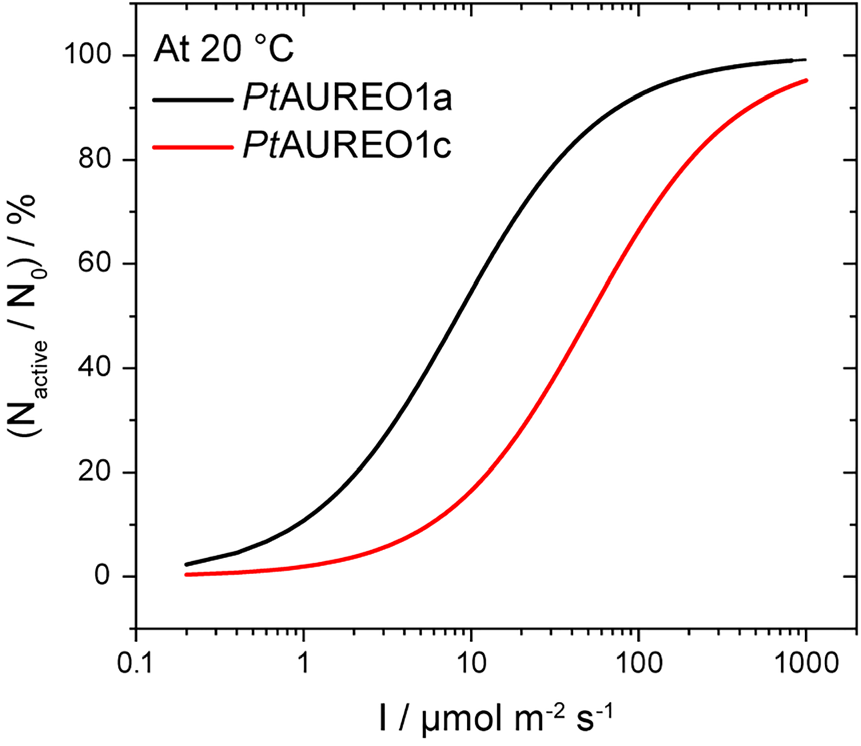
Fraction of activated *Pt*AUREO1a and *Pt*AUREO1c under continuous illumination at different light intensities. The maximal intensity represents full sunlight. Under the assumption that both aureochrome variants have a similar concentration in cells, the percentages of activated photoreceptors were determined by numerical simulations. Information on the quantum yields, the global recovery kinetics, and the integral extinction coefficients were included in the calculations. The differences in response to light point to a role of *Pt*AUREO1c as a high light receptor.

*Pt*AUREO1c and *Pt*AUREO1a are both expressed during the day in the alga^10^ and are therefore available at the same time for light-dependent regulation. The comparative investigation of full-length *Pt*AUREO1c and *Pt*AUREO1a revealed here that these two homologs have clearly different response characteristics, which might justify their simultaneous presence in the alga. Both the quantum yields and the recovery kinetics are different (Fig. 3) and determine the amount of activated receptor present under continuous illumination. The results from SAXS experiments provide us the important information that the recovery rate of the receptor is slower by about a factor of 1.18 than the recovery of the flavin. Other influencing parameters are the integral extinction coefficients that are known and the pigment absorption of the algal cell, which is neglected because it will affect both receptors to the same extent. If we assume a similar concentration of both receptors in the cell, the only parameter modulating the percentage of the activated receptor is the sunlight intensity. A numerical simulation of this dependence clearly illustrates that at full sunlight (model AM1.5g) corresponding to a photon flux density of 1 mmol photons m^−2^ s^−1^, both receptors are equally activated to a large extent, but that *Pt*AUREO1a already responds at much lower intensities to light than *Pt*AUREO1c (Fig. 8). Accordingly, we postulate the role of a high light sensor for *Pt*AUREO1c and a low light sensor for *Pt*AUREO1a rationalizing the evolution of two different subfamilies.

### TR-SAXS experiments on a multidomain full-length photoreceptor

We determined the global structure recovery time of *Pt*AUREO1c by TR-SAXS and compared it to the adduct lifetime obtained by absorption spectroscopy under similar conditions. Even though synchrotron beamlines provide a higher brilliance and therefore a better signal-to-noise ratio at significantly shorter exposure times, we decided to perform the experiments on an in-house setup for the following reasons: (i) The high brilliance may lead to degradation and aggregation of the proteins and therefore influence its kinetics. To circumvent this issue, a continuous flow of fresh sample or an attenuation of the flux can be applied. (ii) Preparation of large sample volumes is time-consuming and expensive. With our homemade sample chamber, 40 *μ*l of sample (between 9.9 mg/ml and 10.8 mg/ml) were sufficient for one kinetic experiment. (iii) The rather long lifetime of the lit state of about 1 h is incompatible with limited beam time allocation at the synchrotron. (iv) We were able to repeatedly observe the dark recovery of an identical subset of proteins over a time range of 24 h, instead of recording consecutive time points in the kinetic on different molecules as performed previously by time-course SAXS experiments.^22^ By this approach, we demonstrated that the structural changes of *Pt*AUREO1c are fully reversible and that even under such extreme conditions, the photoreceptor's stability is little impaired. It should be noted that for the kinetic analysis of photoreceptors, our approach can be directly applied to the scattering curves independent of any modeling.

### Discrepancy between the rate of flavin recovery and of global conformational changes

Extensive studies on *Pt*AUREO1a and truncated constructs consisting of the bZIP-LOV fragment or the isolated LOV domain have strongly contributed to our understanding of the allosteric regulation mechanism in the direction of the signaling pathway.^10–12,14,32^ Much less is known about the reverse reaction upon cleavage of the flavin adduct. Here, we found a discrepancy between the time constant for adduct decay (UV/vis) and the one for the dark structure recovery (SAXS) of full-length aureochrome of several minutes [Fig. 7(a)]. Other studies on the conformational recovery in the back reaction are scarce. Time-resolved nuclear magnetic resonance (NMR) spectroscopy on the isolated LOV2 domain from *Avena sativa* phototropin1 has revealed a difference between the mean time constant resulting from NMR studies and the lifetime of the adduct state by a factor of 1.2, but in the time region of seconds.^38^ Moreover, the recovery of the LOV2-linker from *Arabidopsis* takes place rapidly with a 13 ms time constant according to transient grating experiments.^39^ A difference on the time scale of minutes has only been reported previously for a LOV domain with flanking helices, PpSB1-LOV-R66I from *Pseudomonas putida*, by time-course SAXS experiments.^22^

Commonly, the lifetime of the flavin adduct as determined by UV/vis or fluorescence spectroscopy is equated with the lifetime of the signaling state of the respective LOV protein. Accordingly, it is assumed that the time span required for the conformational changes does not significantly exceed that of the flavin conversion. In the forward reaction, this assumption is supported by experiments on phototropin-LOV2 with transient grating and infrared spectroscopy that show a microsecond to millisecond unfolding of the flanking helices after the microsecond adduct formation.^40,41^ Similarly, the bZIP domain of *Vf*AUREO1 responds with a time constant of 160 ms^9^ by a change in helicity.^10,11^ Rearrangements in the effector domain may even require up to one second as demonstrated by time-resolved X-ray scattering on an artificial LOV-histidine kinase.^19^

But what exactly determines the delay between the recovery of the protein structure and the flavin adduct decay of *Pt*AUREO1c? Previously, two different models were proposed that describe the structural transition from the dark to the lit state of the *Pt*AUREO1a dimer.^10,11^ The first model states that LOV-LOV interactions are apparent in the dark. Upon illumination, the helices that flank the LOV core unfold, and a LOV-LOV rearrangement is induced, resulting in a shortening of the bZIP helices.^10^ In this model, the recovery rate would depend on folding and unfolding processes. However, the folding times of fast-folding proteins range from microseconds to milliseconds^42^ and, commonly, the folding of proteins is completed within seconds.^43^ In contrast, the second model suggests that in the dark, the LOV domains separately interact with the bZIP domains. Upon illumination, a dissociation of the LOV-bZIP interface and a formation of a LOV dimer are triggered.^11^ Accordingly, the dissociation of the LOV-LOV interface and the formation of the LOV-bZIP interface are additional rate-limiting factors for the conformational recovery, which might explain the pronounced discrepancy between the adduct lifetime and the structural recovery time.

Independent of the final structural model, we postulate the presence of an additional intermediate I447 in the photocycle [Fig. 7(b)], which represents a local recovery in the vicinity of the flavin after adduct cleavage but a preservation of global conformational changes of the photoreceptor induced by light. As such, we consider I447 to be a signaling-competent intermediate prolonging the activated state beyond the lifetime of the adduct.

## CONCLUSIONS

Aureochromes are blue light photoreceptors in algae but have also been discussed as versatile candidates for optogenetic applications because of their inverted domain topology compared to other LOV proteins.^44^ The *Vf*AUREO1-LOV domain has indeed been exploited successfully as a tool within fusion proteins in human cell lines^45,46^ and in zebrafish.^47^ Moreover, the application of truncated aureochromes consisting of LOV and bZIP domains as a module to control protein activities was proposed.^48^ For the purpose of an optogenetic device, knowledge about the quantum yield, the full reversibility of structural changes, and the “real” lifetime of the activated state is mandatory.

Here, we characterized the photosensitivity and the recovery kinetics of full-length *Pt*AUREO1c and demonstrated by TR-SAXS that the global recovery rate in the dark is clearly different from that of the flavin switch. Accordingly, we postulate the existence of an additional intermediate I447 in the photocycle (Fig. 7). Future modeling on SAXS data might help to reveal the structural nature of I447. It has been demonstrated that DNA binding and the presence of the N-terminal extension have a moderate influence on the flavin recovery.^10^ Now that the procedure for in-house TR-SAXS has been established, influences of these and other factors on the kinetics of I447 need to be studied. Finally, the fundamental differences in quantum yields and photocycle kinetics of *Pt*AUREO1c as compared to *Pt*AUREO1a strongly suggest roles *in vivo* as high light and low light sensors, respectively.

## SUPPLEMENTARY MATERIAL

See the supplementary material for a size exclusion chromatogram of the sample, an indication for radiation-induced damage at the synchrotron, the determination of quantum yields, the comparison between SVD components, the average relative deviation between reconstructed and experimental scattering data, the check for the integrity of the sample via UV/vis spectroscopy, a comparison of different fits to the SAXS-derived kinetics, and a table summarizing details on SAXS experiments and data analysis.
